# Maternal Vascular Adaptation in High-Risk Pregnancies: Effects of Early Smoking Cessation on Hemodynamic and Endothelial Function

**DOI:** 10.3390/ijms26125781

**Published:** 2025-06-16

**Authors:** Kaltrina Kutllovci Hasani, Mila Cervar-Zivkovic, Ursula Hiden, Adam Saloň, Manurishi Nanda, Bianca Steuber, Katharina Eberhard, Patrick De Boever, Christina Stern, Karoline Mayer-Pickel, Nandu Goswami

**Affiliations:** 1 Department of Obstetrics and Gynaecology, Medical University of Graz, Auenbruggerplatz 14, 8036 Graz, Austria; 2 Vascular Biology Center, Augusta University, Augusta, GA 30912, USA; 3 Gravitational Physiology and Aging Research Unit, Division of Physiology and Pathophysiology, Otto Löwi Research Center of Vascular Biology, Immunity and Inflammation, Medical University of Graz, Neue Stiftingtalstrasse 6/V, 8010 Graz, Austria; 4 Core Facility Computational Bioanalytic, ICAL University of Graz, Stiftingtalstraße 24/1, 8010 Graz, Austria; 5 Research, Innovation & Valorisation Antwerp (RIVA), Antwerp University Hospital, Campus Middelheim, Middelheimlaan 1, 2020 Antwerp, Belgium; 6 Centre for Space and Aviation Health, Mohammed Bin Rashid University of Medicine and Health Sciences, Building 14, Dubai Healthcare City, Dubai P.O. Box 505055, United Arab Emirates

**Keywords:** cardiovascular adaptation, high-risk pregnancy, preeclampsia, smoking cessation, vascular function

## Abstract

Cardiovascular adaptation is vital for a healthy pregnancy but may be impaired in women at high risk for preeclampsia (PE), a condition marked by endothelial dysfunction. Smoking may lower the PE risk but harms vessels, and the effects of early cessation remain unclear. This prospective cohort study assessed vascular changes in high-risk pregnancies and the potential influence of early smoking cessation. Of 110 women screened for PE in the first trimester, 43 were classified as high-risk: 18 former smokers and 25 lifelong non-smokers. Vascular assessments were performed at 11–16, 24–28, and 34–37 weeks of gestation. Parameters included the carotid–femoral pulse wave velocity (cfPWV), asymmetric dimethylarginine (ADMA), mean arterial pressure (MAP), systolic and diastolic blood pressure (SBP, DBP), heart rate (HR), and retinal vessel calibers (central retinal arteriolar and venular equivalents (CRAE, CRVE)). Serum cotinine confirmed abstinence in former smokers. Across gestation, ADMA (*p* = 0.034), MAP (*p* = 0.001), SBP (*p* = 0.033), DBP (*p* = 0.004), and HR (*p* = 0.004) increased, while CRAE (*p* = 0.016) and CRVE (*p* = 0.004) narrowed in late pregnancy; cfPWV remained stable (*p* = 0.783). Non-smokers showed increases in their ADMA (*p* = 0.020), MAP (*p* = 0.001), and DBP (*p* = 0.0001) with no differences between groups. High-risk pregnancies showed vascular changes with similar profiles in former and non-smokers, underscoring the need for broader studies.

## 1. Introduction

Physiological adaptations during pregnancy include reductions in blood pressure and the total peripheral resistance as well as an increase in cardiac output, changes that are critical for ensuring adequate placental perfusion and optimal fetal growth [1,2]. In pregnancies complicated by preeclampsia (PE), these normal adaptations are disrupted [1]. PE, which affects 2–8% of pregnancies worldwide, typically manifests after 20 weeks of gestation with new-onset hypertension, proteinuria, maternal organ dysfunction, or placental insufficiency [1,2,3].

The underlying pathophysiology of PE is complex and not completely understood but is primarily linked to impaired placental development and resultant endothelial dysfunction [2]. Inadequate trophoblast invasion and insufficient spiral artery remodeling lead to placental hypoperfusion, which in turn triggers systemic endothelial dysfunction through the release of placental-derived factors [4]. This dysfunction is marked by an imbalance of vasoactive substances, diminished nitric oxide bioavailability, increased immune activation, and systemic inflammation, all contributing to the vascular complications observed in PE [4,5,6].

The early identification of women at high risk for PE is vital for timely intervention. First-trimester screening that integrates maternal risk factors with biochemical markers has significantly improved risk stratification [7,8]. Prophylactic low-dose aspirin is recommended for high-risk women, as it has been shown to reduce the incidence of early-onset PE [8]. Despite these advances, routine cardiovascular monitoring during pregnancy is limited, typically relying on brachial blood pressure measurements [3,6]. A more comprehensive assessment, including measures of arterial stiffness, wave reflection, and detailed hemodynamic indices, may improve the prediction and management of PE [9,10]. In this context, non-invasive assessments, such as the carotid–femoral pulse wave velocity (cfPWV) for assessing arterial stiffness [9], the measurement of endothelial biomarkers like asymmetric dimethylarginine (ADMA) [11], retinal microvascular imaging, the central retinal arteriolar equivalent (CRAE), and the central retinal venular equivalent (CRVE) [4], offer promising avenues for a more nuanced understanding of vascular adaptations during pregnancy. Assessing the retinal microvasculature is supported by findings that visual disturbances occur in up to 25% of individuals affected by preeclampsia, exemplifying the transient yet potentially severe complications of the condition. Severe vision loss is rare and typically resolves postpartum [4,5].

Cigarette smoking is a well-established risk factor for endothelial dysfunction, vascular inflammation, atherosclerosis, and thrombosis [12,13,14,15] and is linked to adverse pregnancy outcomes [16,17,18,19]. However, little research has focused on whether high-risk pregnancies following pre-pregnancy smoking cessation still exhibit vascular impairment. Addressing this gap, our study aims to evaluate cardiovascular alterations in high-risk pregnancies while comparing vascular profiles between former smokers and lifelong non-smokers. We hypothesized that markers of arterial stiffness, endothelial dysfunction, and retinal microvascular parameters would deteriorate over the course of pregnancy in high-risk women and that former smokers would exhibit a residual vascular impairment compared to non-smokers, reflecting the long-term effects of smoking despite cessation. This research will provide a comprehensive understanding of cardiovascular adaptations in high-risk pregnancies and the potential for vascular recovery following smoking cessation, ultimately guiding more effective preventive and monitoring strategies for high-risk pregnancies.

## 2. Results

### 2.1. Study Population

Between 11 + 0 and 13 + 6 weeks of gestation, a total of 110 pregnant women underwent first-trimester screening for preeclampsia risk. Based on the screening results, 45 were classified as high-risk. Following the exclusion of two pregnancies due to fetal anomalies, 43 women were enrolled in the study for a longitudinal follow-up.

Participants were assessed at three gestational stages: early pregnancy (Visit 1, V1: 11–16 weeks), mid-pregnancy (Visit 2, V2: 24–28 weeks), and late pregnancy (Visit 3, V3: 34–37 weeks). Due to scheduling limitations, not all participants completed all study visits. Measurements were omitted if the participant presented outside the predefined gestational window.

Hemodynamic assessments, including the mean arterial pressure (MAP), systolic and diastolic blood pressure (SBP, DBP), heart rate (HR), and carotid–femoral pulse wave velocity (cfPWV), were available for 33 women at V1. Follow-up completion rates were approximately 70% at V2 and 45% at V3. Serum asymmetric dimethylarginine (ADMA) concentrations were measured in all 43 women at V1 and in 74% and 63% of participants at V2 and V3, respectively. Cotinine was measured at V1 in all participants to biochemically confirm smoking cessation. Retinal microvascular parameters (central retinal arteriolar equivalent [CRAE] and central retinal venular equivalent [CRVE]) were obtained from 29 participants at V1, with corresponding follow-up rates of 79% at V2 and 55% at V3.

The detailed information on parameter-specific sample sizes at each time point is presented in Supplementary Table S1.

Baseline demographic and clinical characteristics are summarized in Table 1. The mean maternal age was 33.2 ± 4.4 years, and the mean body mass index (BMI) was 26.4 ± 5.9 kg/m^2^. Most women (86.0%) conceived spontaneously, while 14.0% conceived via in vitro fertilization. At delivery, the mean gestational age was 38 ± 2 weeks, and the average birth weight was 3108 ± 574 g. Nulliparity was observed in 37.2% of the cohort. Based on the self-reported smoking history, 18 participants who ceased smoking between weeks 5 and 8 of gestation were classified as former smokers, while 25 were lifelong non-smokers. The cohort was predominantly Caucasian (97.7%), with one participant identified as Black (2.3%).

Pregnancy outcomes included preeclampsia (18.6%), gestational diabetes mellitus (14.0%), and gestational hypertension (14.0%), while 53.5% of participants experienced no complications.

### 2.2. Longitudinal Changes in Vascular Parameters by Smoking Status

Within the high-risk cohort, 18 women were classified as former smokers and 25 as lifelong non-smokers. Longitudinal analyses using mixed-effects models (REML) revealed differential trends across the gestation in former smokers and non-smokers. Among former smokers, none of the measured parameters showed statistically significant changes across the three visits, although the heart rate (HR, *p* = 0.057) and systolic blood pressure (SBP, *p* = 0.177) showed modest variability. In contrast, non-smokers exhibited statistically significant increases in several parameters: ADMA levels (*p* = 0.020), mean arterial pressure (MAP, *p* = 0.001), diastolic blood pressure (DBP, *p* = 0.0001), and a borderline trend in HR (*p* = 0.050). The retinal vessel calibers (CRAE, CRVE) and carotid–femoral pulse wave velocity (cfPWV) remained stable over time in both groups (Table 2).

### 2.3. Comparative Vascular Profiles of Former Smokers and Non-Smokers

To further assess whether early smoking cessation influences vascular function, cardiovascular parameters were compared between former smokers and lifelong non-smokers across pregnancy (descriptive data in Supplemental Table S2). At baseline (V1), both groups showed comparable demographic characteristics, including their age (*p* = 0.312) and BMI (*p* = 0.579). No statistically significant differences were observed between former smokers and non-smokers for any cardiovascular parameter at V1, V2, or V3.

**Figure 1:**
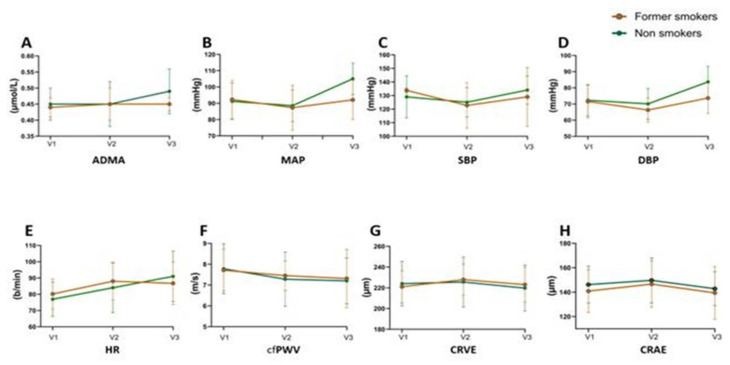
Hemodynamic and vascular parameters in former smokers and non-smokers during pregnancy. Longitudinal changes are shown in (**A**) ADMA, (**B**) MAP, (**C**) SBP, (**D**) DBP, (**E**) HR, (**F**) cfPWV, (**G**) CRVE, and (**H**) CRAE across early (V1, 11–16 weeks), mid (V2, 24–28 weeks), and late (V3, 34–37 weeks) pregnancy. Green lines represent non-smokers; orange lines represent former smokers. Data are presented as mean ± SD. Group comparisons at each visit were assessed using unpaired *t*-tests; significance was set at *p* <  0.05. Similar profiles were observed between groups at all time points (descriptive data with *p* value shown in Supplementary Table S2) without statistical significant differences. Abbreviations: ADMA, asymmetric dimethylarginine; MAP, mean arterial pressure; SBP, systolic blood pressure; DBP, diastolic blood pressure; HR, heart rate; cfPWV, carotid–femoral pulse wave velocity; CRAE, central retinal artery equivalent; and CRVE, central retinal vein equivalent.

Across gestation, both groups showed parallel trends in their asymmetric dimethylarginine (ADMA), mean arterial pressure (MAP), systolic and diastolic blood pressure (SBP, DBP), heart rate (HR), carotid–femoral pulse wave velocity (cfPWV), and retinal vessel calibers (CRAE, CRVE). However, non-smokers exhibited a more pronounced increase in their MAP and DBP between mid- and late pregnancy (MAP: from 88.5 ± 9.7 to 105 ± 9.7 mmHg; DBP: from 70.1 ± 9.5 to 83.7 ± 9.7 mmHg), although this did not reach a statistical significance in group comparisons (MAP: *p* = 0.160; DBP: *p* = 0.066). All other parameters remained statistically similar between groups at each time point (Figure 1).

### 2.4. Longitudinal Changes in Vascular and Hemodynamic Parameters

Progressive and statistically significant changes in vascular and hemodynamic parameters were observed throughout gestation in this high-risk pregnancy cohort, despite some participants missing follow-up assessments due to gestational timing constraints.

**Figure 2:**
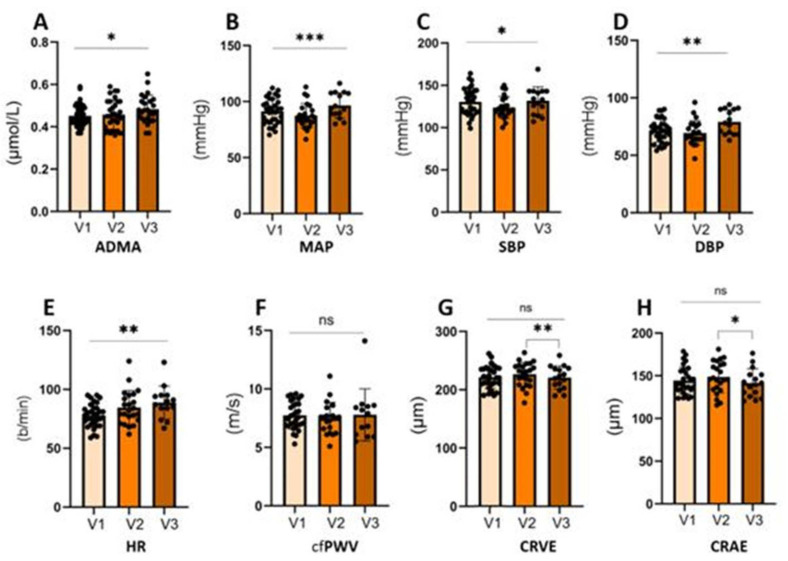
Longitudinal changes in vascular and hemodynamic parameters in high-risk pregnancies across early (V1), mid (V2), and late (V3) gestation. Shown are plasma ADMA (**A**), MAP (**B**), SBP (**C**), DBP (**D**), HR (**E**), cfPWV (**F**), CRVE (**G**), and CRAE (**H**). ADMA and blood pressure parameters (MAP, SBP, DBP) increased progressively over gestation, while retinal vessel calibers (CRVE, CRAE) decreased significantly in late pregnancy. cfPWV remained unchanged. Data are presented as mean ± SD; individual values are shown as black dots. Statistical analysis was performed using mixed-effects models (REML) with Geisser–Greenhouse correction. Tukey’s test was applied for post hoc comparisons. *p* < 0.05 = *; *p* < 0.01 = **; *p* < 0.001= *** and ns = not significant (descriptive data with *p* value shown in Supplementary Table S1). Abbreviations: ADMA, asymmetric dimethylarginine; MAP, mean arterial pressure; SBP, systolic blood pressure; DBP, diastolic blood pressure; HR, heart rate; cfPWV, carotid–femoral pulse wave velocity; CRVE, central retinal vein equivalent; CRAE, central retinal artery equivalent; and REML, restricted maximum likelihood.

Serum asymmetric dimethylarginine (ADMA) levels increased significantly across pregnancy, suggesting a gradual decline in endothelial function (*p* = 0.034; Figure 2A). The mean arterial pressure (MAP) exhibited a biphasic trend, with an initial decline followed by a significant increase in late pregnancy (*p* = 0.001; Figure 2B). A similar pattern was noted for systolic blood pressure (SBP), which decreased mid-pregnancy and rose again toward term (*p* = 0.033; Figure 2C). The diastolic blood pressure (DBP) remained relatively stable early on but showed a significant elevation in the third trimester (*p* = 0.004; Figure 2D). The heart rate (HR) demonstrated a consistent upward trend across all visits (*p* = 0.004; Figure 2E).

In contrast, the carotid–femoral pulse wave velocity (cfPWV) remained stable over time, indicating no significant change in arterial stiffness during pregnancy (*p* = 0.783; Figure 2F).

The retinal vessel analysis revealed late gestational narrowing. The central retinal arteriolar equivalent (CRAE) initially increased slightly, then declined significantly in late pregnancy (*p* = 0.016; Figure 2H). The central retinal venular equivalent (CRVE) followed a similar course, with a late decrease reflecting an increased retinal microvascular resistance (*p* = 0.004; Figure 2G).

A complete overview of sample sizes and descriptive data for each parameter is provided in Supplementary Table S1.

## 3. Discussion

This study presents a comprehensive longitudinal evaluation of the maternal vascular adaptation in high-risk pregnancies, with a specific focus on differences between former smokers and lifelong non-smokers. We investigated changes in the endothelial function, hemodynamic indices, arterial stiffness, and retinal microvascular parameters longitudinally across gestation and examined whether the early pregnancy smoking cessation influences these cardiovascular trajectories.

While both groups (former smokers and lifelong non-smokers) were classified as high-risk, the lifelong non-smokers demonstrated significant increases in the mean arterial pressure (MAP), diastolic blood pressure (DBP), and asymmetric dimethylarginine (ADMA) across gestation, changes that may reflect a typical or even exaggerated hemodynamic adaptation associated with the increased cardiovascular demand during pregnancy.

On the other hand, vascular parameters in former smokers remained comparatively constant over the same time frame. The clear patterns point to possible variations in vascular adaptation, even though no statistically significant results by time interactions or between group differences were found. Rather than signifying a better vascular profile, the relative stability seen in former smokers may be due to the long-lasting effects of previous tobacco exposure on the endothelial function.

According to earlier studies, endothelial and hemodynamic alterations, such as increases in ADMA and the MAP, are typical during pregnancy, especially in women who are at risk for hypertensive disorders [20,21,22,23,24]. Despite their high-risk status, the lack of these alterations in former smokers may indicate a lingering vascular modulation after quitting. Furthermore, it is impossible to completely evaluate the direct vascular effects of continuous tobacco use because our cohort did not include women who smoked during pregnancy.

We found progressive increases in the ADMA, blood pressure parameters, and heart rate throughout gestation in the overall high-risk cohort, which is consistent with endothelial dysfunction and accumulating cardiovascular strain. These results are consistent with earlier studies showing that elevated ADMA levels during pregnancy are a sign of endothelial stress [20,22]. On the other hand, the cfPWV stayed constant during pregnancy, indicating a preserved arterial compliance, which could be the result of compensatory mechanisms, like an increased cardiac output or systemic vasodilation [25,26]. According to the retinal microvascular analysis, the CRAE and CRVE narrowed in late pregnancy, indicating increased microvascular resistance. These trends are consistent with those observed in hypertensive pregnancies [27].

Reference values from the literature provide important context, even though our study lacked a comparator group of normotensive, low-risk pregnancies. With only a slight trimester-based variation, the cfPWV normally falls between 5.5 and 7.0 m/s in healthy nulliparous pregnancies [28]. Depending on the gestational age, blood pressure readings typically fall between 95 and 144 mmHg systolic and 55 and 95 mmHg diastolic [29]. Over the course of three trimesters, retinal vessel calibers stay constant, with the CRAE ranging from 140 to 145 µm and the CRVE from 220 to 225 µm [27,30]. Healthy pregnancies have ADMA levels that progressively increase from a mean of 0.51 µmol/L in the first trimester to 0.58 µmol/L in the third. In contrast, preeclampsia-complicated pregnancies have consistently higher levels, reaching 0.68 µmol/L by late gestation, with the most notable difference occurring in the second trimester [31,32].

These normative ranges support the interpretation of our findings, suggesting that the observed vascular changes reflect deviations consistent with a high-risk pregnancy profile, even in the absence of a healthy control group.

There are a few limitations to take into account. First, we did not include a healthy, low-risk control group, which restricts the interpretation of whether the observed patterns are unique to high-risk pregnancies. Second, we did not gather comprehensive data on the smoking intensity or exposure to secondhand smoke, nor did we have baseline vascular measurements before quitting. In addition, passive smoking, known to affect the arterial stiffness and pulse wave analysis [33], was not assessed in this study. Although our study population consisted exclusively of women, sex-specific differences in vascular responses to smoking are well documented [34] and should be acknowledged when interpreting group-based trends. Future studies should employ multi-center designs and include active smokers to fully elucidate these vascular adaptations. Third, the missing data due to the loss to follow-up, particularly at later gestational stages, may have introduced a bias despite the robustness of mixed-effects modeling. Important confounding variables that may affect the vascular function, including stress levels, socioeconomic status, diet, and physical activity, were also not taken into account.

## 4. Materials and Methods

### 4.1. Study Sample

This prospective cohort study enrolled high-risk pregnant women between November 2019 and September 2022 at the Department of Obstetrics and Gynecology, Medical University of Graz. All participants provided written informed consent, and this study was conducted in accordance with the Declaration of Helsinki. Ethical approval was granted by the local Ethics Committee (reference number 31-541 ex 18/19), and this study was registered on ClinicalTrials.gov (NCT06645340).

Participants were identified as high-risk for developing PE based on first-trimester screening performed between 11 + 0 and 13 + 6 weeks of gestation [35]. Inclusion criteria were a high-risk classification based on screening, a history of PE, daily administration of 150 mg aspirin, and age ≥18 years. Women with twin pregnancies, fetal anomalies, chronic kidney disease, or those who discontinued aspirin were excluded. Participants were further stratified by smoking history into former smokers (those who quit upon pregnancy confirmation) and non-smokers. Smoking cessation was verified by serum cotinine levels.

### 4.2. Vascular and Hemodynamic Measurements

**Figure 3:**
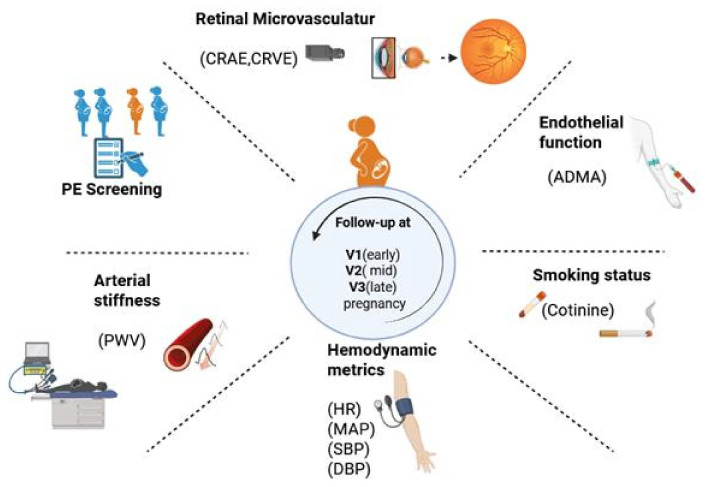
The schematic overview of the study design and assessments.

Participants underwent three evaluation visits: early (11–16 weeks, V1), mid (24–28 weeks, V2), and late pregnancy (34–37 weeks, V3). To ensure standardization, subjects were instructed to avoid physical exertion and stimulants for 48 h before assessments. Overview of the study flow is illustrated in Figure 3.

High-risk pregnancies were identified via routine preeclampsia (PE) screening at 11 + 0 and 13 + 6 weeks of gestation and enrolled following informed consent and study familiarization. Participants were followed longitudinally at three time points: early pregnancy (V1, 11–16 weeks), mid-pregnancy (V2, 24–28 weeks), and late pregnancy (V3, 34–37 weeks).

At each visit, vascular and hemodynamic parameters were assessed, including arterial stiffness (carotid–femoral pulse wave velocity, cfPWV), blood pressure indices and heart rate (MAP, SBP, DBP, HR), retinal microvascular calibers (CRAE, CRVE), and endothelial function (asymmetric dimethylarginine, ADMA). Serum cotinine levels were measured to assess smoking status. Illustration created using BioRender, (www.biorender.com (accessed on 15 April 2025)).

The study protocol included several measurements.

#### 4.2.1. Screening for Preeclampsia

Performed between 11 + 0 and 13 + 6 weeks of gestation, maternal risk factors (age, weight, height, chronic conditions) and biophysical markers (blood pressure, mean arterial pressure [MAP], Pregnancy-Associated Plasma Protein-A [PAPP-A], Placental Growth Factor [PlGF], and uterine artery Doppler pulsatility index) were assessed; however, these routine clinical screening values were not included in the research dataset and are not reported in the present study. These variables were incorporated into the Fetal Medicine Foundation (FMF) algorithm to identify women at high risk for early-onset PE [22]. Participants who did not meet the high-risk threshold were classified as low-risk and excluded from the study. High-risk participants received daily 150 mg aspirin until 36 weeks of gestation [8].

#### 4.2.2. Arterial Stiffness

Arterial stiffness was assessed using cfPWV measured by the VICORDER® system (SMT medical GmbH & Co. KG, Würzburg, Germany) [36]. After a 10 min rest in a semi-supine position, pressure cuffs were placed at the carotid artery and the upper right thigh, and the distance between these sites was recorded. The cfPWV was calculated automatically in meters per second (m/s), reflecting the speed of the pressure wave generated by cardiac contraction traveling along the arterial tree.

#### 4.2.3. Hemodynamic Parameters

Blood pressure and heart rate (HR) were measured twice during cfPWV assessment, and the average values were used to compute MAP using the formula:

MAP = DBP + (SBP − DBP)/3.

#### 4.2.4. Retinal Microvasculature

Retinal images were acquired with a non-mydriatic digital retinal camera (Optomed Aurora, Optomed Oy, Oulu, Finland). Images centered on the optic disc were analyzed with the semi-automated MONA REVA software, version 2.1.1 developed at VITO (Boeretang, Belgium) [37]. The software algorithm segmented retinal vessels and applied post-processing techniques, including double thresholding, blob extraction, and hole filling, to automatically measure the diameters of retinal arterioles and venules located 0.5 to 1 disc diameter from the optic disc margin. Vessel diameters, including the central retinal arteriolar equivalent (CRAE) and central retinal venular equivalent (CRVE), were calculated in micrometers (µm) using the six largest vessels with the revised Parr–Hubbard formula [38].

#### 4.2.5. Biochemical Markers

Blood samples were collected and centrifuged at 800 rpm for 10 min without anticoagulants, and the resulting serum was stored at −80 °C. These samples were obtained to measure serum ADMA [11] and cotinine, to confirm recent tobacco exposure [39]. ADMA levels were determined using an ELISA kit from Immunodiagnostic AG (Bensheim, Germany), and cotinine was measured with a kit from Abnova (KA0930, Taipei, Taiwan), with a detection threshold set to differentiate between non-smokers, passive smokers, and active smokers.

### 4.3. Statistical Analysis

A priori sample size calculation based on differences in arterial stiffness [40] indicated that a minimum of 32 participants (16 former smokers and 16 non-smokers) were required to achieve 90% power at a 0.05 significance level for repeated measures across three time points, assuming a moderate effect size (f = 0.3), as determined using G*Power 3 [41]. Each participant underwent assessments at three gestational stages: early pregnancy (V1: 11–16 weeks), mid-pregnancy (V2: 24–28 weeks), and late pregnancy (V3: 34–37 weeks). Vascular parameters measured included cfPWV, ADMA, MAP, SBP, DBP, HR, CRAE, and CRVE. Data normality was verified using the Shapiro–Wilk test (*p* > 0.05) and Q–Q plots. Group comparisons were conducted using independent *t*-tests or Mann–Whitney U tests, as appropriate. For longitudinal analyses, mixed-effects models with Restricted Maximum Likelihood Estimation (REML) and the Geisser–Greenhouse correction were employed; Tukey’s test was used for post hoc comparisons. Due to challenges in longitudinal follow-up, sample sizes varied across time points, as not all participants completed each visit. Analyses were performed on available cases without imputation. Sensitivity analyses confirmed that the missing data did not introduce systematic bias. Mixed-effects models were applied, which are robust to unbalanced datasets under the assumption that data are missing at random (MAR). All tests were two-sided, with statistical significance set at *p* < 0.05. Data analyses were conducted using SPSS version 27.0, and figures were generated using GraphPad Prism version 9.

## 5. Conclusions

High-risk pregnancies demonstrate progressive endothelial and hemodynamic changes across gestation, with comparable vascular profiles between former smokers and lifelong non-smokers. Future studies should include active smokers, low-risk pregnancies, and detailed assessments of environmental and lifestyle exposures to fully elucidate the vascular impact of smoking history in pregnancy.

## Figures and Tables

**Table 1:** Baseline characteristics of the high-risk pregnancy cohort.

Characteristic	Value *n*/% (means ± SD)
Participants (*n*)	43
Age (years)	33.2 ± 4.4
BMI (kg/m^2^)	26.4 ± 5.9
Gestational age at delivery (wk.)	38 ± 2
Birth weight (grams)	3108 ± 574
Nulliparous patients, *n* (%)	16 (37.2%)
Previous smokeres/non-smokeres, *n*	18/25
Ethnicity, *n* (%)	
Caucasian	42 (97.7%)
Black	1 (2.3%)
Conception mode, *n* (%)	
Spontaneous	37 (86%)
IVF	6 (14%)
Outcome, *n* (%)	
Preeclampisa (PE)	8 (18.6%)
Gestational dieabetes mellitus (GDM)	6 (14%)
Gestational hypertention (GH)	6 (14%)
No complications	23 (53.5%)

Data are presented as mean ± standard deviation (SD) or as number (*n*) with corresponding percentages (%), as appropriate. Baseline characteristics including age, BMI, ethnicity, and mode of conception were recorded at enrollment. Pregnancy outcomes, including gestational age at delivery, birth weight, and complications, preeclampsia (PE), gestational diabetes mellitus (GDM), and gestational hypertension (GH), were documented at delivery.

**Table 2:** Longitudinal vascular and hemodynamic parameters across gestation in former smokers and non-smokers.

Parameter	Stage	Former Smokers (*n* = 18) *n*/% (means ± SD)	P (V1 vs. V2 vs. V3)	Non-Smokers (*n* = 25) *n*/% (means ± SD)	P (V1 vs. V2 vs. V3)
ADMA (μmol/L)	V1	18/100 (0.44 ± 0.03)	0.231	25/100 (0.45 ± 0.05)	0.020
	V2	12/67 (0.45 ± 0.05)		20/80 (0.45 ± 0.07)	
	V3	9/50 (0.45 ± 0.02)		18/72 (0.49 ± 0.07)	
HR (b/min)	V1	13/72 (80.18 ± 9.2)	0.057	20/80 (76.95 ± 10.5)	0.050
	V2	9/50 (88.00 ± 11.6)		14/56 (84.0 ± 15.3)	
	V3	7/34 (86.71 ± 13.0)		8/32 (91.00 ± 15.5)	
MAP (mmHg)	V1	13/72 (92.3 ± 11.7)	0.236	20/80 (91.2 ± 11.2)	0.001
	V2	9/50 (87.3 ± 13.7)		14/56 (88.5 ± 9.7)	
	V3	7/34 (92.1 ± 12.1)		8/32 (105 ±9.7)	
SBP (mmHg)	V1	13/72 (133.8 ± 1.78)	0.177	20/80 (129.0 ± 15.5)	0.186
	V2	9/50 (122.7 ± 16.8)		14/56 (125.1 ± 10.8)	
	V3	7/34 (129.0 ± 21.8)		8/32 (134.1 ± 10.4)	
DBP (mmHg)	V1	13/72 (71.58 ± 10.13)	0.234	20/80 (72.3 ± 9.7)	0.0001
	V2	9/50 (66.3 ± 7.4)		14/56 (70.1 ± 9.5)	
	V3	7/34 (73.7 ± 9.5)		8/32 (83.7 ± 9.7)	
CRAE (µm)	V1	10/56 (140.9 ± 17.5)	0.527	19/76 (146.2 ± 15.1)	0.058
	V2	9/50 (146.6 ± 19.0)		14/56 (149.7 ± 18.3)	
	V3	6/34 (139.4 ± 21.6)		10/40 (142.8 ± 13.9)	
CRVE (µm)	V1	10/56 (221.0 ± 15.6)	0.462	19/76 (224.0 ± 21.6)	0.186
	V2	9/50 (227.9 ± 14.8)		14/56 (225.6 ± 24.2)	
	V3	6/34 (223.0 ± 16.7)		10/40 (219.7 ± 22.2)	
cfPWV (m/s)	V1	13/72 (7.72 ± 1.0)	0.568	20/80 (7.78 ± 1.2)	0.4370
	V2	9/50 (7.45 ± 0.7)		14/56 (7.28 ± 1.3)	
	V3	7/34 (7.31 ± 1.4)		8/32 (7.20 ± 1.1)	

Data are shown as the mean ± SD at each visit (V1: 11–16 weeks; V2: 24–28 weeks; and V3: 34–37 weeks). A statistical analysis was performed using mixed-effects models with a restricted maximum likelihood (REML) estimation to assess differences across visits within each group. *p*-values indicate the significance of the change across timepoints (V1 vs. V2 vs. V3) within each group. A *p* < 0.05 was considered statistically significant. CRAE = central retinal arteriolar equivalent; CRVE = central retinal venular equivalent; cfPWV = carotid–femoral pulse wave velocity; ADMA = asymmetric dimethylarginine; MAP = mean arterial pressure; SBP = systolic blood pressure; DBP = diastolic blood pressure; and HR = heart rate.

## Data Availability

The datasets generated from the current study are available from the corresponding author on request.

## Supplementary Materials

The following supporting information can be downloaded at https://www.mdpi.com/article/10.3390/ijms26125781/s1.
