# Supplementary material for: Maternal Vascular Adaptation in High-Risk Pregnancies: Effects of Early Smoking Cessation on Hemodynamic and Endothelial Function
Source: Int J Mol Sci. 2025 Jun 16;26(12):5781. doi: 10.3390/ijms26125781 (PMC12193101; doi:10.3390/ijms26125781)
Supplement: Supplementary file 1 [file ijms-26-05781-s001.zip › ijms-3654060-supplementary.pdf]

Supplementary Material

Supplemental Tables S1. Descriptive hemodynamic and vascular parameters across pregnancy in the high-risk cohort.

Supplemental Tables S2. Descriptive cardiovascular parameters in former smokers and non-smokers across pregnancy.

Supplemental Tables S1. Descriptive hemodynamic and vascular parameters across pregnancy in the high-risk cohort.

**Parameter**	**V1** (11–16 wk.) n/% (means ± SD)	**V2** (24–28 wk.) n/%(means ± SD)	**V3** (34–37 wk.) n/%(means ±SD)	**P**
**ADMA** (µmol/L)	43/100(0.45 ± 0.04)	32/74(0.45 ± 0.07)	27/63(0.48 ± 0.06)	**0.034**
**MAP** (mmHg)	33/100(91.2 ± 11.3)	23/70(87.5 ± 10.9)	15/45(96.6 ± 11.3)	**0.001**
**SBP** (mmHg)	33/100(130.6 ±15.9)	23/70(123.8 ± 12.8)	15/45(131.7± 16.2)	**0.033**
**DBP** (mmHg)	33/100(71.58 ± 9.9)	23/70(69.4 ± 10.4)	15/45(79.0 ± 10.6)	**0.004**
**HR** (bpm)	33/100(77.9 ± 9.9)	23/70(84.6 ± 14.3)	15/45(88.8 ± 13.9)	**0.004**
**cfPWV** (m/s)	33/100(7.72 ± 1.1)	23/70(7.48 ± 1.3)	15/45(7.79 ± 2.2)	0.783
**CRAE** (µm)	29/100(144.3 ±15.9)	23/79(148.5 ± 18.2)	16/55(141.5 ±16.6)	**0.016**[a]
**CRVE** (µm)	29/100(223.2± 19.5)	23/79(226.5 ± 20.7)	16/55(221 ±19.8)	**0.004**[a]

Data are presented as mean ± standard deviation (SD) along with the number of participants (n) and the percentage relative to the baseline sample for each parameter.

V1: early pregnancy (11–16 weeks); V2: mid-pregnancy (24–28 weeks); V3: late pregnancy (34–37 weeks).

Repeated measures were analyzed using mixed-effects models with Restricted Maximum Likelihood Estimation (REML) and the Geisser–Greenhouse correction. Post hoc comparisons were performed using Tukey's test. Bold values indicate statistical significance ($p < 0.05$).

[a] p-value indicates a significant difference at late pregnancy.

Abbreviations:ADMA, asymmetric dimethylarginine; MAP, mean arterial pressure; SBP, systolic blood pressure; DBP, diastolic blood pressure; HR, heart rate; cfPWV, carotid–femoral pulse wave velocity; CRAE, central retinal artery equivalent; CRVE, central retinal vein equivalent.

Supplemental Tables S2. Descriptive cardiovascular parameters in former smokers and non-smokers across pregnancy.

**Parameter**	**Stage**	**Former smokers** (n=18) n/% (means ± SD)	**Non-smokers** (n=25) n/% (means ± SD)	**p-value**
**Age** (years)	V1	18/100 (31.79 ± 5.39)	25/100 (33.30 ± 3.64)	0.312
**BMI** (kg/m²)	V1	18/100 (27.34 ± 6.78)	25/100 (26.26 ± 5.92)	0.579
**ADMA** (μmol/L)	V1	18/100 (0.44 ± 0.03)	25/100 (0.45 ± 0.05)	0.430
	V2	12/67 (0.45 ± 0.05)	20/80 (0.45 ± 0.07)	0.811
	V3	9/50 (0.45 ± 0.02)	18/72 (0.49 ± 0.07)	0.193
**HR** (b/min)	V1	13/72 (80.18 ± 9.2)	20/80 (76.95 ± 10.5)	0.400
	V2	9/50 (88.00 ± 11.6)	14/56 (84.0 ± 15.3)	0.516
	V3	7/34 (86.71 ± 13.0)	8/32 (91.00 ± 15.5)	0.585

**MAP** (mmHg)	V1	13/72 (92.3 ± 11.7)	20/80 (91.2 ± 11.2)	0.809
	V2	9/50 (87.3 ± 13.7)	14/56 (88.5 ± 9.7)	0.810
	V3	7/34 (92.1 ± 12.1)	8/32 (105 ±9.7)	0.160
**SBP** (mmHg)	V1	13/72 (133.8 ± 1.78)	20/80 (129.0 ± 15.5)	0.452
	V2	9/50 (122.7 ± 16.8)	14/56 (125.1 ± 10.8)	0.660
	V3	7/34 (129.0 ± 21.8)	8/32 (134.1 ± 10.4)	0.567
**DBP** (mmHg)	V1	13/72 (71.58± 10.13)	20/80 (72.3 ± 9.7)	0.844
	V2	9/50 (66.3 ± 7.4)	14/56 (70.1 ± 9.5)	0.354
	V3	7/34 (73.7 ± 9.5)	8/32 (83.7 ± 9.7)	0.066
**CRAE** (µm)	V1	10/56 (140.9 ± 17.5)	19/76 (146.2 ± 15.1)	0.410
	V2	9/50 (146.6 ± 19.0)	14/56 (149.7 ± 18.3)	0.697
	V3	6/34 (139.4 ± 21.6)	10/40 (142.8 ± 13.9)	0.704
**CRVE** (µm)	V1	10/56 (221.0 ± 15.6)	19/76 (224.0 ± 21.6)	0.701
	V2	9/50 (227.9 ± 14.8)	14/56 (225.6 ± 24.2)	0.804
	V3	6/34 (223.0 ± 16.7)	10/40 (219.7 ± 22.2)	0.764
**cfPWV** (m/s)	V1	13/72 (7.72 ± 1.0)	20/80 (7.78 ± 1.2)	0.897
	V2	9/50 (7.45 ± 0.7)	14/56 (7.28 ± 1.3)	0.755
	V3	7/34 (7.31 ± 1.4)	8/32 (7.20 ± 1.1)	0.882

**Cotinine**<br><br>(ng/mL)	V1	-	-	na

Data are presented as mean ± standard deviation (SD) along with the number of participants (n) and the percentage relative to the baseline sample for each parameter.

Pregnancy stages: **V1** (early: 11–16 weeks), **V2** (mid: 24–28 weeks), **V3** (late: 34–37 weeks).

Group comparisons between former smokers and non-smokers were conducted using unpaired t-tests; $p < 0.05$ was considered statistically significant.

"–" indicates values below the detection limit; "n.a." = not applicable.

Abbreviations: ADMA, asymmetric dimethylarginine; MAP, mean arterial pressure; SBP, systolic blood pressure; DBP, diastolic blood pressure; HR, heart rate; cfPWV, carotid-femoral pulse wave velocity; CRAE, central retinal artery equivalent; CRVE, central retinal vein equivalent.
